# Recent Advancements in Smart Biogenic Packaging: Reshaping the Future of the Food Packaging Industry

**DOI:** 10.3390/polym14040829

**Published:** 2022-02-21

**Authors:** Vandana Chaudhary, Sneh Punia Bangar, Neha Thakur, Monica Trif

**Affiliations:** 1 College of Dairy Science and Technology, Lala Lajpat Rai University of Veterinary and Animal Sciences, Hisar 125001, India; dhakavandana18@gmail.com; 2 Department of Food, Nutrition and Packaging Sciences, Clemson University, Clemson, SC 29634, USA; 3 Department of Livestock Products Technology, Lala Lajpat Rai University of Veterinary and Animal Sciences, Hisar 125001, India; 4 CENCIRA Agrofood Research and Innovation Centre, 400650 Cluj-Napoca, Romania; monica_trif@hotmail.com

**Keywords:** biogenic, smart packaging, biodegradable, material, food

## Abstract

Due to their complete non-biodegradability, current food packages have resulted in major environmental issues. Today’s smart consumer is looking for alternatives that are environmentally friendly, durable, recyclable, and naturally rather than synthetically derived. It is a well-established fact that complete replacement with environmentally friendly packaging materials is unattainable, and bio-based plastics should be the future of the food packaging industry. Natural biopolymers and nanotechnological interventions allow the creation of new, high-performance, light-weight, and environmentally friendly composite materials, which can replace non-biodegradable plastic packaging materials. This review summarizes the recent advancements in smart biogenic packaging, focusing on the shift from conventional to natural packaging, properties of various biogenic packaging materials, and the amalgamation of technologies, such as nanotechnology and encapsulation; to develop active and intelligent biogenic systems, such as the use of biosensors in food packaging. Lastly, challenges and opportunities in biogenic packaging are described, for their application in sustainable food packing systems.

## 1. Introduction

Growing anxieties concerning the ecological impact of waste, carbon footprint, consumer inclination towards ready-to-eat foods with enhanced shelf life, and the sustainability of fossil fuels have all fueled a surge in scientific collaboration to develop or find alternatives to traditional food packaging materials. Moreover, conventional packaging materials for food are estimated to exceed 700 million annually and are expected to reach one billion by the end of 2021 [1]. As a result, the plastic industry is the prime source of plastic pollution, which is expected to increase two fold by 2050 [2]. Food packaging industries are increasingly inclined towards biogenic plastics or biopolymers made from renewable resources, to solve the environmental challenges and limited supplies of petroleum-based polymers. Biogenic plastics are made from renewable raw resources that can be regenerated through natural processes.

Smart biogenic packaging is an innovative, swiftly emerging concept, where sustainability and real-time monitoring of food are coupled together, ensuring safe and healthy food, alongside commercial and ecological prosperity. Smart biogenic packaging integrates active and intelligent packaging solutions to provide consumers with more reliable information about food product conditions. It also generates a shielding effect for the food by incorporating active substances such as antimicrobial agents in a biogenic polymer matrix [3]. Biosensors can play a vital role in detecting food pathogens, harmful additives, or allergens. These sensors are composed of a bioreceptor that detects a biochemical signal and a transducer that transforms this into a quantitative response [4]. Various companies are now producing biosensors to identify specific pathogens in food samples, to avert unpleasant situations and assure food safety. To get highly precise and sensitive results, microbial whole-cell sensors, nucleic acid, and bacteriophage-based sensors can be used. Multiple techniques of incorporating biosensors in food packets are described subsequently in this article.

A vast amount of literature has been published on the impact of petro-based packaging materials on the environment. Hence, it has become pertinent to evaluate the various biogenic polymers, their properties, prerequisites, and deviances, to raise awareness about green packaging; thereby, encouraging its prominence. This review is expected to empower the food industry and regulatory authorities to deploy this unique, clean, and environmentally friendly approach on a large scale.

## 2. Transformation from Conventional Packaging to Biogenic Packaging

Plastics are frequently used for packing because they are light, inexpensive, and adaptable to many applications. However, due to the low biodegradability of these petroleum-based polymers, environmental issues have been growing [5]. Polyolefins and their replacements, polyesters, and polyamides are the three main types of conventional packaging materials used in the food and food product industries. Polymers such as polypropylene (PP), low-density polyethylene (LDPE), linear low-density polyethylene (LLDPE), polyvinyl chloride (PVC), polyvinylidene chloride (PVDC), polystyrene (PS), and oriented polystyrene (OPS) are examples of polyolefins and their alternatives. Water bottles are mostly polyesters, such as polyethylene terephthalate (PET) and other aliphatic and aromatic polyesters. PAs are typically used in films or trays for food products that are extremely sensitive to oxygen. The majorities of these components are not biodegradable and will accumulate in landfills over time, causing environmental harm. Even though numerous recycling techniques exist, packing materials are frequently contaminated with leftover food, making recycling cumbersome and consequently unviable. Environmental awareness and strict environmental restrictions are driving research into alternatives to food packaging materials, and attempts are being made at both academic and industry level to incorporate bioplastics into various consumer items [6]. Although plastic packaging has proven to be workable, the production of petroleum-based plastics emits greenhouse gases (particularly CO_2_), and their disposal results in plastics ending up in landfills, becoming refuse in land and water streams, and eventually contaminating the waterways, due to a lack of collection or careful management [7]. The food packaging sector is trying to determine how to make plastic more environmentally responsible.

Additionally, food-packaging companies and the food industry have been attempting to replace old, nonrenewable petroleum-based materials with abundant, low-cost, renewable, and biodegradable alternatives, to become more sustainable. The overall question is how can plastic become (more) sustainable? In this context, the food-packaging producers and food industries have been using abundant, low-cost, renewable, and biodegradable alternatives to the traditional, nonrenewable petroleum-based resources, such as bioplastics [8].

As far as consumer behavior is concerned, eco-friendliness has become the fashion of the day, thanks to the growing popularity of worldwide environmental protection and the notion of sustainable development. As such, the logistics industry pays more attention to green packaging. Although most customers lack specific knowledge of green packaging, they have demonstrated a considerable willingness to pay for it. Furthermore, consumers would place a higher value on the usability of green packaging, such as accessibility, renewability, and preventative abilities, compared to the costs and aesthetics of green packaging [9]. Another notable variation in consumer behavior toward plastic use is the rise in popularity of ready-to-eat foods, particularly in urban regions, where modern lives tend to limit the available time. Understanding the pro-environmental behavior of convenience food customers is particularly difficult, since there is frequently a conflict between eco-friendly intentions and the time limits imposed by modern city living [10].

Due to the strong demand from industry, new bio-based polymers and bio-based conventional polymers are predicted to drive rapid growth in research and development over the next decade. Global reliance on petrochemical-based materials and their environmental consequences has increased the strain on nonrenewable resources. As a result, sustainable alternatives are preferred, since they are significantly greener, relatively clean, degradable, reusable, and functional after use [11]. It is noteworthy that, although progress has been made in the development of alternative packaging methods, there is still no perfect solution that can meet all of the sustainability criteria, while also fulfilling the primary role of food packaging: to keep and transport the packaged items in good condition [12].

Evaluating the long-term viability of food packaging necessitates a more holistic approach that considers various factors. This should include the use of materials that, among other things, produce no greenhouse gas emissions, can be recycled or reused, generate no landfill trash, consume less water, are created with renewable energy, do not pollute the air, and do not impair human health. Although progress has been made in the development of alternative packaging methods, there is still no perfect solution that can meet all of the sustainability criteria and, in the end, fulfills the function of food packaging: to store and transport the packed goods in good condition [13].

## 3. Biogenic Packaging Polymers in Food Packaging-Types

Biogenic packaging is a new generation of packaging, which is garnering worldwide recognition due to its environmental amenability and biodegradability. Biogenic polymers or resins are used to make artificial or organic processed macromolecules into sustainable packaging materials from bio-based (agricultural and marine) sources, and which are biodegradable and/or recyclable [14,15]. Biogenic polymers are associated with the notion of sustainability and also exhibit a lower carbon dioxide (CO_2_) footprint, contrary to traditional packaging materials. New biogenic polymers are continuously evolving, with a plethora of properties [16] to help relieve concerns about the exhaustion of fossil reserves and the global warming exacerbated by the use of petrochemicals.

**Figure 1:**
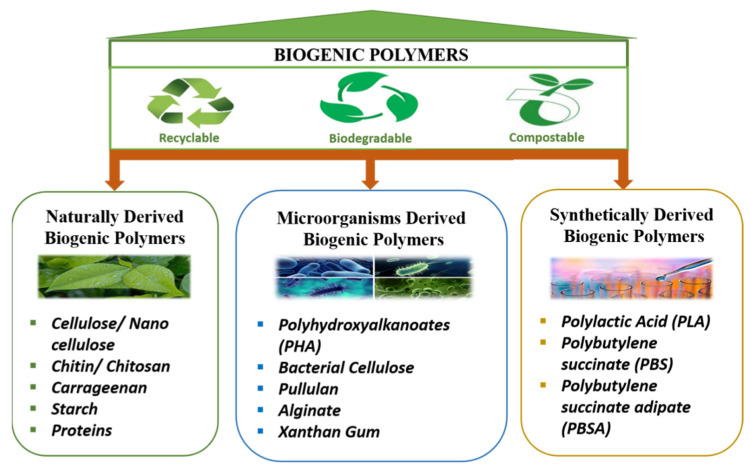
Schematic categorization of biogenic polymers.

Technological innovations for converting these naturally derived resources into value-added chemicals and innovative polymerization methods for producing superior quality, cheap polymers with configurable frameworks and functionalities are critical components of long-term development. In addition, the present need is to develop ultra-modern and cutting-edge techniques for unfolding their internal arrangement and facilitating their use in advanced sectors, such as in biogenic sensors [17]. Based on their origin, biogenic polymers are schematically grouped under three classes (Figure 1): the first encompasses the polymers derived naturally from biomass, the second class includes polymers biosynthesized by microflora, and the third is synthetic biogenic polymers, made from bioderived monomers.

### 3.1. Naturally Derived Biogenic Polymers

Sustainable polymers are derived from easily procurable and biocompatible materials such as biomass rather than traditional fossil fuels such as oil and gas, especially through biological and biochemical processes (Table 1). Researchers have made substantial efforts to synthesize novel, natural biogenic polymers that are chemically equivalent to, and that can replace or outperform, petroleum-based polymers [18]. Table 1 encompasses several naturally derived biogenic polymers, with their compositional units and properties.

#### 3.1.1. Cellulose/Nano-Cellulose

Cellulose is an organic, sustainable, structural polysaccharide composed of D-glucose monomers. Cellulose can be derived from various bio-sources such as wood, cotton, hemp, and agricultural by-products, etc. [19]. Aside from paper manufacturing, cellulose without alteration has very few applications, particularly in the food packaging industry. The enzymatic, chemical, or mechanical modification of cellulose leads to the generation of carboxymethyl cellulose, methyl cellulose, and hydroxypropyl cellulose, which can be judiciously used as a coating material for food packaging applications. Composite films are made from cellulose acetate, chitosan, and silica. Enhancement in tensile strength (TS) and reduction in oxygen transmissibility have been reported.

Furthermore, the hydrophilic inclination of the film, due to the presence of chitosan was overcome using silica, by tuning it to be hydrophobic, which is a desirable property in food packaging [20]. Compared to commercially available polyethylene, the cellulose-based film fabricated from delignified banana stem fibers by Ai et al. [21] demonstrated higher gas and moisture permeability. The increased permeability aided ethylene release, thereby delaying the ripening of bananas and extending the storage period of mangoes.

Nanocellulose is an ideal material for food packaging, because of its excellent rigidity (comparable to that of polyethylene terephthalate) and lower oxygen portability (equivalent to that of ethylene vinyl alcohol). Nanocomposites are considered an effective alternative to improve the effectiveness of the polymers above to a level suitable for food packaging applications [22]. Numerous research studies have reported that using nanostructured cellulose fibers as a strengthening material in composite packaging can result in significant improvements in gas permeation properties, thermal stability, and biodegradability [23]. Bio-based films derived from cellulose nanofibrils and oil showcased excellent ductability and stability at temperatures up to 300 °C [24]. Ghaderi et al. [25] illustrated that nanocomposites formed from cellulose nanofibers extracted from sugarcane bagasse with polylactic acid ameliorated the water vapor permeability. Pan et al. [26] reported that fish gelatin film, when reinforced with microcrystalline cellulose, had higher tenacity and elasticity values than pure films. At the same time, the elongation at break was lower.

#### 3.1.2. Chitin/Chitosan

Chitin is the second most abundant natural polymer after cellulose, is derived from exoskeletons of crustaceans, and has emerged as a promising alternative to petroleum-based packaging materials in the food packaging industry [27]. Chitin’s composition is identical to cellulose, except for having an acetamide group on the alpha carbon atom instead of the secondary hydroxyl group in the cellulose molecule. It is utilized for the generation of chitosan through deacetylation by an alkali [28].

Chitosan is a soluble form of chitin and is applied in the packaging industry, due to its low cost and abundant natural availability [29]. The properties of chitosan are mainly dependent on the degree of acetylation [30]. The existence of a non-polar acetyl group imparts hydrophobic characteristic properties to chitosan [31]. Chitosan-based packaging films have been shown to exhibit fairly good mechanical properties and are also less permeable to gases. However, natural polymers showcase a higher affinity for moisture, thereby illustrating increased water vapor permeability [32]. A variety of strategies have been used to optimize the characteristics and properties of immaculate polymers by the amalgamation bioactive substances or blending them with other natural biopolymers. Laksmanan et al. [33] ascertained that the pores existing in a blend of chitosan and microbial-derived extracellular polymeric substances allowed a continuous exchange of gases, while minimizing moisture transfer, making it more suitable for food packaging. The moisture, barrier, mechanical, and optical properties of chitosan and rice starch film improved by exposing it to ultrasonic treatment. It was found that due to the internal formation of cross-linkages by rice starch, the seepage of water in the composite films was hindered. In addition, TS and elongation at break also improved [34].

Bioplastics articulated from the consolidation of chitosan, montmorillonite, and ginger essential oil showed good oxygen barrier properties, retarding oxidation in foods with unsaturated fats [35]. It was reported that the embodiment of eugenol-loaded chitosan nanoparticles in thermoplastic flour upgraded the moisture barrier tendency and exhibited superior antioxidant activity [36]. Collagen has captured experts’ interest as a possible synthetic polymer alternative. Chitosan-based composite films demonstrated outstanding thermal stability, compatibility, and adhesion [37]. Ahmed and Ikram [38] testified that biodegradable chitosan and gelatin biocomposite packaging films had an increased TS, ultra-violet barrier properties, and decreased water vapor permeability. As a result, it is reasonable to believe that chitosan, when coupled with proteins/carbohydrates, essential oils, and other ingredients, could provide a variety of pre-programmed properties to give bio-based packaging desirable attributes.

#### 3.1.3. Carrageenan

Carrageenans are high molecular weight biopolymers obtained from the Rhodophyceae family of seaweed cell walls. These are water-soluble, extremely flexible, linear sulfated galactan polysaccharides, with spiral helical structures found in the cavities of the cellulose network in plants [39,40]. These spiral helical confirmations are capable of producing many types of gels at ambient temperature. They are typically found in two forms: native, and degraded. On a commercial scale, carrageenan is used as an additive in the food processing industry, as stabilizers, gelling agents, thickeners, etc. However, owing to its inherent properties, it is also used as the base material for the production of bio-based packaging materials [41]. In addition, bio-nano-composite films of carrageenan have an enhanced biodegradability index, which can mitigate environmental impacts [42].

Although the packaging generated from carrageenan has good a gas barrier capability, it has reduced water resistance characteristics; thereby, hampering its usage in food packaging [43]. As a result, the carrageenan matrix is frequently intermingled with different polymers, to optimize the barrier properties. It was observed that when kappa-carrageenan is blended with polyvinyl alcohol, a positive modification in water vapor transmissibility, TS, bursting ability, and water solubility of the film was produced [44]. Similarly, Martiny et al. [45] demonstrated a significant reduction by 54 percent in water vapor permeability of carrageenan films imbibed with olive leaves extract; in addition, the formed films were more flexible. In agreement with the previous study, a steady refinement was noticed in the mechanical properties and water vapor permeability of Ipomoea batatas and kappa-carrageenan blended films [46]. Similar results were also obtained by Sedayu et al. [47] in the case of carrageenan and nanocellulose composite films.

#### 3.1.4. Starch

Starches are widely available polysaccharides and one of the most affordable groups of biodegradable polymers. They are also referred to as hydrocolloid biopolymers. Biopolymers are made from various starches, including rice, potato, corn, cassava, tapioca, and others [48]. Due to the firm configuration of polysaccharide molecules, they block the diffusion of oxygen and carbon dioxide gases. However, these bio molecules are prone to water transmission through films; their fragile nature and lack of mechanical stability have led to the concept of coupling with lipids or other biopolymers to counter these limitations [49]. Composite biofilms made with cassava, pinhão thermoplastic starch, compostable polyester poly butylene adipate co-terephthalate, green tea, and rosemary extracts helped improve water vapor permeability [50]. A progression in flexibility, water vapor resistance, and TS was perceived in a starch-based film reinforced with cellulose nanofibers [51]. Similar results were remarked by Ali et al. [52] in starch films with polysaccharide-based crystals. Moreover, the Young’s modulus and protection from UV rays produced an augmentation in TS. With the addition of salicylic acid to the starch matrix, improvements in TS and impediment of water vapor permeability were observed. In addition to this, the films had good activity against *S. aureus* and *B. subtilis* [53].

#### 3.1.5. Proteins

Proteins employed for the formation of films are derived from renewable sources and are easily degradable compared to their plastic counterparts. Protein macromolecules comprise precise amino acid sequences joined by amide linkage and molecular arrangement that can be degraded by proteases [54]. They are frequently used as film-forming substances. Proteins possess an additional benefit in their amphiphilic nature, besides electrostatic charge and denaturation properties [55]. Various changes can be brought about in the secondary, tertiary, and quaternary structure of proteins to suit the needs of film-forming substances. These variations can be made using heat, irradiation, chemical, mechanical treatment, pressure, and enzymatic applications.

Proteins originating from milk (casein and whey protein), plant sources (soy protein, maize zein), wheat gluten, pea protein, rice bran protein collagen, albumin from eggs, myofibrillar protein of fish, and keratin are among the most frequently utilized proteins in edible film and coating compositions [56]. For instance, chitosan added to rapeseed protein hydrolysate augmented the density, mechanical properties, and TS of the film. Furthermore, it also exhibited antibacterial action against *E. coli*, *B. subtilis*, and *S. aureus* [57,58]. The flexibility, elongation at break, ultra-violet ray blocking capacity of fish gelatin film increased with the incorporation of citric acid [59].

**Table 1:** Properties of naturally derived biogenic polymers used in food packaging.

Biogenic Polymer	Monomeric Unit	Structure of Monomeric Unit	Properties
Cellulose/Nanocellulose	D-glucose	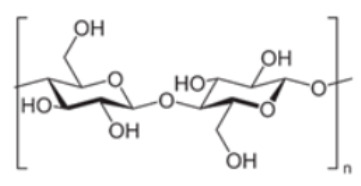	Most abundant biopolymer on earth. Monomers are joined by β-1,4 glycoside bonds. The elementary organization of cellulose is fabricated from micro sized string-like structures microfibers, which are further made up of nanosized microfibrils [60]. Non-toxic nature and exceptional strength to weight ratio marks it a preferred choice for food packaging materials [61].
Chitin	N- acetylglucosamine	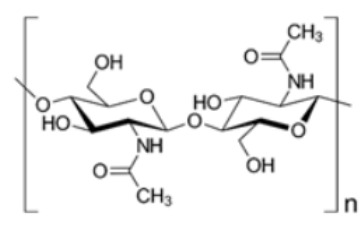	Derived from the exoskeletons of crustaceans. Composition is identical to cellulose, except for an acetamide group on the alpha carbon atom instead of the secondary hydroxyl group in the cellulose molecule. Utilized for the generation of chitosan by a process of deacetylation using an alkali [27].
Chitosan	N-acetyl-D-glucosamine	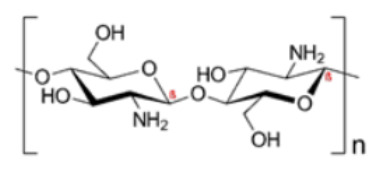	Semicrystalline and easily soluble in organic acids such as malic, lactic, etc. [62]. Biodegradable, nontoxic, biocompatible [28]. Positively-charged, a linear polysaccharide made from β-(1-4)-linked D-glucosamine and N-acetyl-D-glucosamine units [63]. The degree of acetylation may vary from 0 to 70% [29]. Higher affinity for water [32]. Antagonistic activity against bacteria and fungi [64].
Carrageenan	Sulfated d-galactose and l-anhydrogalactose	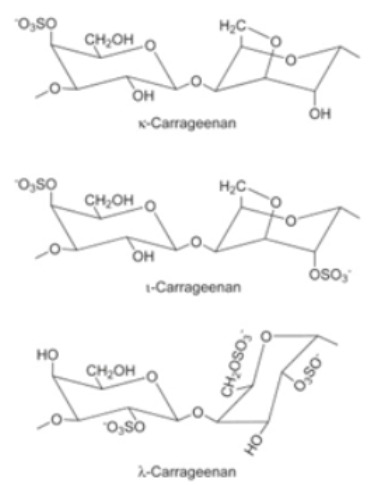	Obtained from the red edible seaweed family also known as Irish Moss [65]. Linear chains of alternating 3-O-substituted β-d-galactopyranosyl units and 4-O-substituted α-d-galactopyranosyl units. It exists in three forms: (i) kappa: ability to produce hard and stiff gels in the companionship of potassium ions, (ii) iota: creates soft gels with calcium ions, (iii) lambda: does not possess gelling properties and is used as a thickening agent. Less resistance to water and exhibits excellent mechanical properties [66] Possesses amazing gelling ability, good film-forming properties, having optimal transparency and TS [67].
Starch	Glucose monomers joined in α 1,4 linkages	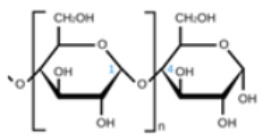	Native starch polymers are tasteless and odorless, semipermeable to gas, water, and flavoring components [30]. Packaging obtained by using starch as a base material is flexible, transparent, environmentally friendly, and cheap [68]

### 3.2. Microorganism-Derived Biogenic Polymers

Microbiota are involved in synthesizing several biogenic polymers with diverse characteristics in food packaging.

#### 3.2.1. Polyhydroxyalkanoates (PHA)

Bacillus sp., Cupriavidus nector, and other bacteria ferment surplus carbon-based feedstocks to generate bio-based, biodegradable, and compostable biopolymers: polyhydroxyalkanoates [69]. PHAs are demarcated by dint of several monomeric carbons in their chains: short chains have three to six carbons, medium-chain lengths include seven to sixteen carbons, and long chains have more than sixteen carbons. The properties of PHA polymers can be customized according to the application, by integrating various polymers into the polymer chain. Aside from chain length variability, many PHAs with different functional groups, such as halogens and aromatic groups, have been successfully fabricated [70]. PHA’s are increasingly being preferred as a substitute for petro-based packaging materials because of their superior thermal stability. PHAs with a medium chain length are used as coatings for cheese and cutlery for serving food [71].

The first PHA to be discovered was Poly-3-hydroxybutyrate (PHB). The most prevalent PHA packing resins are PHB and its copolymer with poly-hydroxy valerate. Only polypropylene and polyethylene can compete with PHB, in terms of mechanical characteristics. Its superior mechanical features, such as high elastic modulus and TS, and excellent moisture and gas barrier capabilities, qualify it for food packaging [72]. Despite some covetable attributes, its practical industrial application is limited due to its (i) innate aging due to secondary crystallization, which leads to brittleness over time [73]; (ii) sluggish crystallization results in the formation of big spherulites, causing high fracturability [74]; (iii) thermally instable [75], and (iv) high manufacturing cost [76].

#### 3.2.2. Bacterial Cellulose

Bacterial cellulose has garnered worldwide attention because of its exceptional physical and chemical characteristics, such as biocompostibility, being environmentally friendly, marked mechanical properties, low cost of production, and hydrophilic nature [77]. Bacterial cellulose is a linear and unbranched microbial polymer, generated as an exopolysaccharide by certain bacteria such as Acetobacter, Gluconacetobacter, Sarcina, Agrobacterium, etc. Unlike plant cellulose, it lacks pectin, lignin, and hemicellulose, which makes its isolation and purification process simpler and requiring of less energy input compared to plant-derived cellulose, which includes the usage of toxic chemicals [78]. It comprises ultrafine nanofibrils that materialize into a three-dimensional mesh-like structure, further stabilized by inter and intra-molecular hydrogen bonding [79]. The high fiber content, degree of crystallinity, and TS provides it with an edge compared to plant cellulose [80]. Scientists have made tremendous efforts to develop highly-functional bacterial cellulose composites with tailor-made characteristics [81].

Reviewing the literature showed that in situ and ex situ processes can be harnessed to functionalize bacterial cellulose [82]. The most prevalent procedure for biopolymer composite synthesis is the in situ approach, which involves first adding reinforcing elements to the culture medium, such as agar, sodium alginate, starch, montmorillonite carboxymethylcellulose, and so on. This method’s superiority is due to its simplicity. Furthermore, the additional compounds become an intrinsic component of the 3D fibril structure, giving the composite stability and favorable properties. A major constraint of this process is that these added polymers may be insoluble in culture media and may potentially hinder bacterial growth [83].

On the other hand, ex situ modification is based on the bacterial cellulose production process. In this post-production process, bioactive chemicals are injected into a porous, nanofibrillar bacterial cellulose matrix. The abilities to employ bioactive chemicals and preserve the natural structure of bacterial cellulose are the most important aspects of this approach. One of the downsides is that only nanoparticles can gain entry to the bacterial cellulose pores [84]. Owing to the chemical reactivity of bacterial cellulose, due to hydroxyl groups, many permutations and combinations are feasible for fulfilling the requirements of the food packaging industry.

An improvement in tensile and barrier characteristics was observed when bacterial cellulose in combination with silver nanoparticles was dispersed in chitosan nanocomposite films [85]. Light and mechanical properties and biodegradability were also augmented by combining bacterial cellulose with carboxymethylcellulose [86]. Bacterial cellulose bio-based packaging is one of the fastest-growing trends in the food sector, with benefits for the natural environment, human health, and the quality of stored food goods. However, several hurdles remain for low-cost commercial production of BNC-based packaging materials, such as the insubstantial yield of known bacterial nanocellulose strains and the relatively high operational expenses (e.g., expensive culture medium, bioactive agents), particularly when compared to synthetic alternatives.

#### 3.2.3. Pullulan

Pullulan is an edible, linear, unbranched, non-ionic, water-soluble, non-mutagenic, and commercially accessible exopolysaccharide generated from the fermentation medium *Aureobasidium* pullulans resembling yeast. Pullulan can be processed into an odorless, thin, tasteless, and transparent packaging material [87]. It comprises maltotriose monomeric units linked together by α-(1-6)-glycosidic bonds. The single linkage pattern governs its characteristic features of flexibility, elasticity, and solubility [88,89], whereas hydroxyl groups are responsible for its barrier properties [90]. It has low viscosity in comparison to other polysaccharides, does not gel, and has strong oxygen barrier qualities in films and coatings; in addition, it has good durability in aqueous solutions over a wide pH range [91]. However, its use in food packaging applications is limited by its high cost. As a result, pullulan is intermingled with other biopolymers to reduce costs and improve its material properties.

The development of pullulan-based composite films reinforced by zinc oxide nanoparticles and propolis intensified UV blocking capacity, improving mechanical strength by 25 percent. Furthermore, this alloying helped in optimizing its water vapor permeability. In addition, the composite film produced a very good action against *L. monocytogenes* and *E.coli* [92]. Cinnamon essential oil at 12% and Tween 80 added to a pullulan matrix complimented the action against food pathogens and antioxidant activity of the blended film. However, a decrement in water permeation, TS, and transparency was observed [93].

Another study by Luís et al. [94] reported that pullulan and apple fiber films had better TS and elasticity at *p*-value <0.05 than pure pullulan films. Moreover, a boost in hydrophobicity was reported. They also could scavenge free radicals, reduce lipid peroxidation, and stop the growth of the recognized foodborne pathogens *S. aureus*, *L. monocytogenes*, *B. cereus*, *E. coli*, *P. aeruginosa*, *S. typhimurium*, and *E. faecalis*. Pullulan alkyl esters were synthesized with varying degrees of substitution and carboxylic anhydrides. Films made from pullulan esters demonstrated maximal barrier efficiency against oxygen and moisture, proving their suitability for extending the shelf life of packed food products [95]. Bionanocomposite packaging developed by intermixing pullulan with cellulose nanofibers showed an improved tenacity by 60 percent and thermal stability. Water vapor and oxygen transmission rates diminished by 32 and 38 percent, respectively, with the addition of cellulose nanofibers [19]. Its proven safety record as a nature-friendly and biocompatible biopolymer has gained widespread regulatory recognition. As per the United States Food and Drug Administration, pullulan falls under the ‘generally regarded as safe (GRAS)’ category [96].

#### 3.2.4. Alginate

Alginates are structural polysaccharides obtained from brown algae (*Phaeophyceae*) and bacteria such as *Pseudomonas* and *Azotobacter*. Alginate is composed of monomeric unit consisting of (1,4)-linked -D-mannuronic acid (M) and -L-guluronic acid (G) residues [97]. The various combinations of M and G blocks produce at least 200 different alginates [98]. Alginates have low toxicity, compatibility with living organisms, are environmentally friendly, and have superior film-forming ability. However, they are highly hydrophilic, leading to an amplification in water vapor transmission rate [99]. An amalgamation of copper sulfide nanoparticles at 0.5 percent in alginate-based blend films improved mechanical, UV blocking, and hydrophobic properties. These films exhibited decent action against *E. coli* and *L. monocytogenes* [100]. In a similar study, the imbibition of thymol in alginate-based film increased elongation at break, TS, and UV barrier characteristics. However, reduced solubility in water, water permeation, and swelling ratio were found [101]. Various other biocompatible compounds have been added or incorporated to improve the characteristics of alginate-based packaging: (a) for good mechanical properties: micro fibrillated cellulose and calcium chloride; (b) for declining water vapor transmission rate: calcium chloride, (c) for enhancing flexibility, glycerol or sorbitol may be added [102].

#### 3.2.5. Xanthan Gum

Xanthan Gum is a high molecular weight, extracellular polysaccharide synthesized by fermentation of carbohydrates by bacteria *Xanthomonas campestris*. The basic monomeric unit of xanthan gum is D-glucose units with a trisaccharide side chain. Two mannose units of a side-chain are differentiated by guluronic acid. When in contact with water, xanthan gum showcases remarkable endurance against a wide range of pH variations, acid, and alkalis, by a toughening and insulating effect developed by anionic trisaccharide side chains [103]. The composite film obtained by a combination of xanthan gum (4 g per liter), pectin, and sodium alginate exhibited a TS of 29.65 MPa, elongation at break of 19.02 percent, and WVTR 18.12 × 10–11 g/m2.s.pa, proving a better choice for packaging of fresh-cut fruit and vegetables [104]. According to Rukmanikrishnan et al. [105], composite films obtained by the interaction of xanthan gum and agar had good light properties, and were transparent, biocompatible, and more stable at a wide range of temperatures

### 3.3. Synthetically Derived Biogenic Polymers

Polymers are obtained by modifying natural polymers or produced synthetically from synthetic monomers, so that they can degrade naturally without leaving detrimental by-products in the environment such polylactic acid, polybutylene succinate, and so on (Table 2). Synthetic biopolymers have received considerable attention due to their distinctive benefits over natural polymers, in terms of their reliability and versatility for complementing a broad array of applications; as well as, due to their biodegradability and environmental friendliness.

#### 3.3.1. Polylactic Acid (PLA)

PLA is aliphatic polyester with lactic acid as its basic constitutional unit. Lactic acid is produced by fermentation of carbohydrates obtained from corn, wheat, potato, or agricultural wastes, such as whey and molasses [106]. However, corn is the most preferred biomaterial because it serves as an impeccable feedstock for the process of fermentation; thereby, resulting in the generation of pure lactic acid [107]. However, the commercial applicability of PLA is restrained because of its easy fracturability, lower softening temperature, poor water and gas permeation capability, premature aging, and diminished shock tolerance [108]. To overcome these limitations and broaden its applicability, it is recommended to prepare blends or composites of PLA and reinforce them with various fillers [109]. A blended film comprising PLA and lignin was developed. A significant decrease in swelling ratio and stretchability was noticed. However, a substantial advancement was observed in its light barrier characteristics, antimicrobial activity, and compostability. Regarding the environmental conditions, bio-decomposition of PLA typically takes 6–24 months. Silva et al. [110] incorporated kraft lignin at different concentrations in PLA composites to reduce the bio-decomposition time. The researchers concluded that PLA-lignin composites containing 10 percent lignin could significantly reduce bio-decomposition time.

Moreover, these composites had comparable TS to that of pure PLA packaging. Kim et al. [111] proposed incorporating zinc oxide nanoparticles into a PLA film. The composite films offered excellent antagonistic action against *E. coli* and *Staphylococcus aureus*. Moreover, the films demonstrated excellent ultra-violet ray barrier properties.

An amalgamation of lignin in PLA increased the thermal stability, but was responsible for reducing the degree of crystallinity, because lignin inhibits the dexterity of PLA chains during crystallization. Bio-decomposition of PLA-lignin multilayer films may be attributed to the fact that degradation initiates during the amorphous phase and progresses to the crystalline phase [112]. PLA multilayer films with gelatin supplemented with an extract from almond shells showed a lower oxygen gas penetrability. Their mechanical strength was analogous to commercially available plastic packaging materials [113]. Akin to this study, PLA films reinforced with magnesium oxide nanoparticles demonstrated better gas resistance and tensile characteristics, as well as optimized antibacterial effectiveness and UV monitoring competence. Hence, the integration of magnesium oxide nanoparticles in PLA seems to become a very enticing strategy for developing new food packaging materials [114].

PLA fibers display a low aroma withholding capacity and are extremely water-resistant. Besides this, by dint of their fat and oil resistance and good aroma barrier characteristics, they are a widely known precursor for the synthesis of thermoformed containers for food packaging [115]. PLA has a comparable TS and elastic modulus to polyethylene terephthalate (PET), but has a much lower elongation at break [116]. Furthermore, its impact strength is comparable to that of polystyrene (a relatively brittle polymer). Another drawback with PLA food packaging is that it produces a loud noise that consumers perceive as an undesirable property [117]. Zych et al. [118] found that plasticization of PLA with epoxidized soybean oil methyl ester achieved an increase in elongation at a break of nearly 800 percent. Furthermore, these films recorded significantly less noise compared to the packaging material of pure PLA. It was envisaged that by employing different concentrations of PLA coating on soy protein isolate film, transparency, permeation, and strength properties of the film could be increased.

Moreover, PLA is becoming more popular in the catering industry as a substitute for traditional plastics, because it is deemed safe for direct contact with food [119]. Owing to its thermoplastic properties, comparable to traditional synthetic polymers, it is suitable for a wide range of applications in the food and packaging industry. It acts as a good substitute for low-density polyethylene, high-density polyethylene, polystyrene, etc., which are frequently used to fabricate rigid containers, disposable containers, and so on [120,121].

#### 3.3.2. Polybutylene Succinate and Polybutylene Succinate Adipate

Polybutylene succinate (PBS) is a biodegradable aliphatic polyester derived from the poly-condensation reaction of succinic acid and 1,4-butanediol [122]. PBS has various advantages, including being heat proof and having well-balanced mechanical properties, which are useful in a variety of applications [123]. As a result of its superior fat transfer resistance at elevated temperature, PBS is a reasonable choice compared to petroleum-based polymers and perfluorinated chemicals [124]. PBS copolymers were synthesized by mixing with various compounds containing glycol moiety. A reduction in crystallinity was observed, making these copolymers extra flexible [125]. Thurber and Curtzwiler [114] deduced that PBS blends could replace perfluoroalkyl substances and the petro-based polymer packaging used in ready-to-eat foods. A convincing enhancement in TS was recorded in PBS and microfibrillated cellulose composites by Zhou et al. [126]. Xu et al. [127] reported an increase in TS and a decrease in oxygen and water permeation in blends of PBS with nanocrystalline cellulose and chitin whiskers. Films prepared from PBS and polybutylene adipate-co-terephthalate (PBAT) showed a decrement in water and gas permeability.

Moreover, with an increase in the concentration of PBAT, an increment in elongation at break and a more textured surface break was produced [128]. The blow film extrusion method produced composite film of PBS, PBAT, and linear low-density polyethylene (LLDPE). Films with more PBS had diminished water vapor transport property and oxygen gas permeability; thereby, inhibiting fungus growth in packed bread, due to dehydration [129]. By enhancing the concentration of kenaf fiber in PBS, the firmness, durability, and fracture strain of a blended film depreciated, due to inadequate adhesion between the two [130].

Polybutylene succinate adipate (PBSA) is synthesized by adding adipic acid to source materials during PBS synthesis. PBS has a higher crystallinity and is better suited for molding, whereas PBSA has a lower crystallinity and is better suited for film applications. The amalgamation of PBSA with hydrolyzed cellulose produced a packaging with enhanced mechanical properties. It could be successfully employed to produce molded containers to be utilized in agriculture and plant nurseries [131].

**Table 2:** Properties of synthetically-derived biogenic polymers used in food packaging.

Biogenic Polymer	Monomeric Unit	Structure of Monomeric Unit	Properties
*Synthetic Biogenic Polymer*			
Polylactic acid (PLA)/Polylactide	Lactic acid/lactide	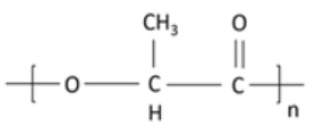	Non-toxic, biodegradable, aliphatic polyester [132]. Produced from lactic acid either by polycondensation reaction or through ring-opening polymerization of lactide monomer [133]. Derived from renewable sources such as starch and/or sugar [134]. Reduced carbon emissions (15% to 60%) and 25% to 55% lower energy consumption in comparison to petroleum-based polymers [135]. Enhanced strength, transparency, fracture strain, and high elastic modulus [136]. Glass transition lies between 50–80 °C, and its crystalline melting temperature varies from 130 and 180 °C [137].
Poly(butylene succinate) (PBS)	Succinic acid and butanediol	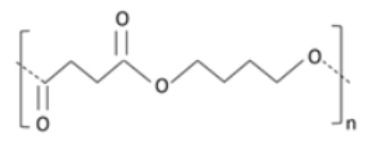	Aliphatic polyester [138]. Polycondensation product of bio-based succinic acid and 1,4 butanediol [139]. Exhibits good elongation properties [140]. Poor gas barrier characteristics [141]. Highly crystalline [142]. Is stiff. High crystallinity results in a reduced rate of degradation, because of its highly-coordinated structure [143].
Polybutylene succinate adipate (PBSA)	Succinic acid, adipic acid and butanediol	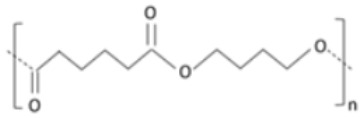	Semi-crystalline polyester produced by co-condensation of succinic and adipate acid with 1-4-butanediol.

## 4. Biogenic Smart Packaging

Active and intelligent packagings are emerging tools for food packaging, ensuring and enhancing food safety. They can be attained either by using active ingredients such as antimicrobials in food, or intelligent indicators such as biosensors to detect contamination or spoilage in food. Active food packaging is the most common, with edible films and coatings found in meat, seafood, fruits and vegetables, and dairy products. Due to detrimental effects on human health and the environment, natural antioxidant and antimicrobial sources, such as natural extracts, are becoming more popular in the packaging research sector as active components in edible film and coating formulations. The intelligent food packaging method employs indicators and sensors embedded in the packaging and monitors changes in the physiological characteristics of the foodstuffs (due to microbial and chemical degradation) [144].

### 4.1. Antimicrobial Biogenic Packaging Using Nanotechnology

Edible films are now increasingly being produced to maintain food quality and increase food safety. Film-forming dietary components, including proteins, polysaccharides, and lipids, are commonly used to create the film matrix. Active compounds, such as antioxidants and antimicrobials, can be added to these composites to improve their functional qualities [145]. Not only should an ideal edible film protect food from pollutants and food-borne diseases, but it should also maintain its structural integrity. Materials generally recognized as safe (GRAS) and that can be eaten with food should be utilized in edible films. Antioxidants, food additives, vitamins, and antibiotic chemicals can all be carried by these films. Antimicrobial agents, such as organic acids, bacteriocins, essential oils, and extracts, are employed to treat edible films. Microbial growth, lipid oxidation, and textural change in Pacific white shrimp were all prevented by essential oil (carvacrol, citral)-containing films [146], as a suitable example. The release of these chemicals into food during preservation improves food safety [147]. Another example is propolis (bee glue) in biodegradable sheets to prevent the fungal spoiling of kashkaval cheese [148].

Furthermore, nanotechnology-based innovations such as bio-nanocomposites and nanoencapsulation technologies have been used to improve the efficiency of complementarity in antimicrobial bio-based packaging technologies [149]. Nanotechnology is a branch of science concerned with materials at the nanoscale (less than 100 nanometers). The surface-to-volume ratio of nanomaterials is high. As a result, these materials are much more reactive than their bulk counterparts. Nanomaterials differ from macroscale materials in terms of their physical and chemical characteristics. We can now make nanoscale edible coatings for packaging perishable food items, such as meat, fruits, and cheese, using nanotechnology and nanomaterials. The packaging can also contain active ingredients that act as an antibacterial and antioxidant covering [150]. The addition of nano-titanium dioxide to TPS-based films by blown extrusion extended the shelf life of packaged bananas, while simultaneously acting as an oxygen scavenger [151]. Moreover, several incorporated antimicrobial agents also enhanced the compatibility between polymer blends, which improved the smoothness of microstructure and films properties, while extending shelf-life; e.g., sodium nitrite [152,153], potassium sorbate, sodium benzoate [154], carvacrol [155].

### 4.2. Biobased Sensors

A biosensor is an analytical device that measures the concentration of a chemical in a sample. Sensors are the most promising technology for future IP system development and enhancement. Detectors are generally divided into three functional sections: sensing, signal conditioning (signal processing), and the interface, which displays the observed attributes. The first part detects physical or chemical qualities and converts them to an electric signal in most cases. The signal conditioning unit processes the resulting signal. This unit amplifies, linearizes, and scales the signal [156]. Sensors can be of numerous types, as classified in Table 3. The most common type is a bioreceptor (enzyme, whole-cell, antibody, aptamer, nucleic acid) linked to an appropriate transducer. The transducer converts the physicochemical or biological signal generated by the precise interaction between the target molecule and the biocomponent into a measured attribute. The choice of bioreceptor and transducer is determined by the sample’s properties and the property to be measured. The bioreceptor is the key component of a biosensor, responding only to a single analyte and not to any interference that may be present in the sample under investigation [157]. The biosensors often give information, such as the degree of freshness of the product packaging, via a color change that can be easily detected by both the food distributor and the consumer. However, most of the indicators presently in use are synthetic materials that are non-renewable and non-biodegradable. As there is a pressing need to increase the sustainability of food packaging, sensor selection should reflect this need [158].

**Table 3:** Classification of various biosensors, along with their key features.

Classification System	Biosensor	Key Features	Reference
Bioreceptors Based	Enzyme biosensors	Enzyme based microreactors are developed that interact with the food environment and detect changes.	[159]
	Antibody biosensors	Antibody layer in the sensor is used to recognize the target, often a pathogenic or spoilage microbe and convert it into a signal.	[160]
	Aptamer biosensors	Aptamers can be defined as a type of oligonucleotides that have high specificity and affinity for the target organisms in food that cause spoilage. Biosensors based on aptamers have great potential as a tool for pathogen detection in food.	[161]
	Whole cell biosensors	Living cells as biosensors offer features such as a easy fabrication process and flexibility of detection stratagems.	[162]
	Nano biosensors	Magnetic nano-sensors can be useful in detecting various residues (such as pesticide, antibiotics), additives (antioxidants) or analytes (bisphenol A, aflatoxins) in food in extremely low quantities.	[163]
Transducer Based	Electrochemical biosensors	They can be further categorized into amperometric, potentiometric, voltammetric, conductometric, and impedimetric. Low cost, ease of operation, portability, simplicity, and easy miniaturization are some of the advantages of electrochemical biosensors. Recent works showed that they work best with two-dimensional nanomaterials, as these enhance the sensitivity, repeatability, and specificity of the electrochemical biosensors.	[164]
	Optical biosensors	This works on the principle of a signal generation proportionate to the concentration of analyte in a sample. They enable screening of a plethora of analytes or compounds and the use nanostructured materials for assessment of optically active materials. They enable smart colorimetric detection, making the food package active and smart. The low cost of fabrication is one of the striking features of optical biosensors.	[165]
	Electronic biosensors	Biosensors that act as electronic tongues or noses have been developed based on pattern recognition principles, and act as freshness indicators for various fruits and vegetables. Observation by the naked eye is a huge advantage.	[166]
	Gravimetric biosensors	They are also known as mass-based biosensors. These produce measurable signals upon detecting a change in mass on the sensor surface.	[167]
	Acoustic biosensors	Acoustic biosensors are based on the ability of the target molecule to bind and vibrate at the frequency of the piezoelectric crystals used in the sensors. The physical attributes of the acoustic waves thus generated are analyzed, and inferences about the analyte and its concentration are drawn.	[168]
Technology-Based	Nano biosensors	Nanomaterials offer great electrochemical, optical, mechanical, magnetic, and conductive properties. Examples include nanowires, quantum dots, and nanotubes that amplify the initial signal and lower detection limits.	[169]
	SRP biosensors	Stimuli-responsive polymers (SRPs) respond to the changes in the food environment or external stimuli such as pH, enzymes, etc., and aid in detecting spoilage in food packaging systems.	[170]
	Chip based biosensors	These act as promising point of care (POC) devices, enabling target detection. Liquid crystal technology is used for the development of chip-based biosensors in food.	[171]
	Electrometers	These come in handy when monitoring the real-time quality or estimating the perishability of food material. The dielectric properties of biopolymers aid in analysis based upon electrical conductivity and electrets state, and the peaks thus obtained are studied.	[172]
Detection system based	Optical biosensors	They ensure food safety owing to their application in POC devices. These sensors are quick, competent, and dependable.	[173]
	Electrical biosensors		
	Electronic biosensors		
	Thermal biosensors		
	Magnetic biosensors		
	Mechanical biosensors		

Recent advances include nanomaterials and nanopolymer-based biosensors that have shown tremendous potential in food safety. Gold nanoparticles provide an excellent platform for developing fast, low-cost, portable, and on-site food safety biosensors. Hydrogen bonding, nucleic acid hybridization, aptamer-target binding, antigen-antibody recognition, enzyme inhibition, and enzyme mimicking activity are all mechanisms used in gold nanoparticle-based biosensors. Foodborne diseases, heavy metals, mycotoxins, pesticides, herbicides, veterinary medications, and illicit additions can be detected using gold nanoparticle-based biosensors [174].

Bio-based materials, such as chitosan hydrogels, have also piqued the curiosity of food technologists worldwide. The advantages of chitosan-based hydrogels include their biocompatibility, stimuli responsiveness, embedding ability, swelling, biodegradability, non-toxicity, low cost, and high bioactivity. Biosensors for food packaging can be made using these properties. CH-based hydrogels have been employed as biosensors in various fields, because they can respond to external stimuli and turn environmental inputs into observable signal outputs by swelling or embedding bioactive chemicals that interact with an input element. Because of their antibacterial, antioxidant, and biodegradability qualities, CH-based hydrogels have a lot of potential in intelligent food packaging systems [175].

Food freshness, food integrity, fruit maturity, food containment, and food monitoring and tracing are all possibilities for applying biosensors in food packaging [165]. It can be rightly said that biosensors are innovative concepts that have only recently been introduced in intelligent packaging. More research is needed to overcome the commercialization obstacles such as their high cost and high technical skill requirements [176].

## 5. Challenges and Future Perspective

Biogenic polymers have progressed as an important topic among the scientific fraternity and industrialists as a green, biodegradable, environment-friendly, novel approach to mitigating the environmental hazards contributed by petro-based packaging. Despite a large number of advantages, biogenic polymers face several hurdles for fabricating viable packaging applications. However, their commercialization is hindered, especially regarding unsatisfactory gas and water permeation, mechanical strength, hydrophilic nature, and thermal attributes. Furthermore, the high cost of production also adds to the disadvantages of biogenic polymers [177]. Slow-growing market demand and acceptance by consumers can also lead to higher production costs. Technical hurdles relating to the functional and production specificities of bio-based materials, which differ significantly from petrochemical plastics, are the principal impediment to commercial adoption.

Customers opting for biogenic polymer-based packaging misunderstand the ‘bio’ designation (i.e., biogenic, bio-based, bioplastic, biodegradable, etc.). They may perceive the word ‘biodegradable’ as a material which can be composted at home. However, the bulk of biogenic polymers-based plastics, for instance, PLA, can only be biodegraded in special composting installations constantly under high temperature and humidity, rendering them unfit for home composting. They take a considerable time to decompose when peppered, thereby causing negative repercussions to the environment [177,178]. Blending, multi-layering, co-extrusion, coating, and nanotechnology have arisen due to these shortcomings. Moreover, extensive research on the array of biogenic polymers must be conducted, to determine an appropriate strategy contributing to the production of superior quality packaging at a justifiable cost. In the world of food packaging, a broad consortium of biopolymers supplemented with a range of bioactive compounds from plant and animal sources could be a viable substitute [179].

Another important aspect to consider while manufacturing bio-based packaging is toxicity. When processing bio-based plastics, various additives such as chain promoters, antioxidants, cross-linking agents, certain catalysts may be added to obtain the desired attributes. Some compounds may not be properly bonded to the polymer matrix, which may migrate to the human food chain through chemical migration [180]. According to Ernstoff et al., 2019, biogenic plastic-based toxicity is usually increased during the production or at the time of degradation [181]. Nonetheless, the majority of biodegradable polymers and biogenic plastics have found employment in the food industry, due to changes in manufacturing methodologies, with risk detection and characterization before large-scale commercial use.

## 6. Conclusions

Biogenic smart packaging has emerged as a solution to the world’s urgent need to lower carbon footprints and ensure food safety. New packaging materials and technologies can be created by developing active and smart bio-based films developed in novel ways. Biogenic films are low-cost, environmentally friendly, biodegradable, and useful. They can be made from a variety of natural sources. Despite advancements in nanotechnology and its subsequent use in developing smart/intelligent biogenic food packaging, commercial manufacturing is constrained by cost, production economics, useful life, biodegradation concerns, toxicity, and appropriate agricultural waste availability. This emphasizes the importance of focusing more on chemical safety when developing truly ‘better’ plastic alternatives. Besides this, the advancement in smart biogenic packaging is influenced by food laws, policies, and legislative reforms, and the global demand for food and energy resources. Hence, more research is needed to refine approaches or develop new methods for boosting the performance of biogenic packaging materials and expanding their use in diverse industries.

## Data Availability

The data that support the findings of this study are available from the corresponding author upon reasonable request.
